# Nitrogen uptake and transfer in a soybean/maize intercropping system in the karst region of southwest China

**DOI:** 10.1002/ece3.3295

**Published:** 2017-09-10

**Authors:** Hao Zhang, Fuping Zeng, Zhigang Zou, Zhenqian Zhang, Youzhi Li

**Affiliations:** ^1^ State Key Laboratory for Conservation and Utilization of Subtropical Agro‐Bioresources Guangxi University Nanning China; ^2^ Key Laboratory of Agro‐Ecological Processes in Subtropical Region Institute of Subtropical Agriculture Chinese Academy of Sciences Changsha China; ^3^ Huanjiang Observation and Research Station for Karst Ecosystem Chinese Academy of Sciences Huanjiang China; ^4^ College of Agriculture Sciences Hunan Agricultural University Changsha China

**Keywords:** crop nutrition, isotopic tracing, karst soil, nitrogen transfer, nitrogen uptake, soybean/maize intercropping

## Abstract

Nitrogen (N) deficiency occurs in over 80% of karst soil of southwest China, which restricts regional agricultural production. To test whether N fixed by legumes becomes available to nonfixing companion species, N fluxes between soybean and maize under no, partial, and total restriction of root contact were measured on a karst site in southwest China. N content and its transfer between soybean and maize intercrops were explored in a 2‐year plot experiment, with N movement between crops monitored using ^15^N isotopes. Mesh barrier (30 μm) and no restrictions barrier root separation increased N uptake of maize by 1.28%–3.45% and 3.2%–3.45%, respectively. N uptake by soybean with no restrictions root separation was 1.23 and 1.56 times higher than that by mesh and solid barriers, respectively. In the unrestricted root condition, N transfer from soybean to maize in no restrictions barrier was 2.34–3.02 mg higher than that of mesh barrier. Therefore, it was implied that soybean/maize intercropping could improve N uptake and transfer efficiently in the karst region of southwest China.

## 1 INTRODUCTION

Intercropping, as the simultaneous cultivation of two or more crops in the same site, had long been used for crop production in time and space (Awal, Koshi, & Ikeda, 2006; Ofosu‐Budu, Noumura, & Fujita, 1995; Yong et al., 2015). Researches on intercropping typically indicate that increased biomass production is a result of improved resource use efficiency of water, nutrients, land, solar radiation, and atmospheric CO_2_ (Antunes, Varennes, Rajcan, & Goss, 2006; Isaac, Hinsinger, & Harm, 2012). Intercropping also could contribute to reducing negative environmental consequences of agroecosystems, such as climate change, soil acidification, terrestrial ecotoxicity, or cumulative energy demand (Gao, Wu, Zhao, & Wang, 2014). Thus, intercropping has been considered to play an important role in the development of sustainable agriculture (Lithourgidis, Dordas, Damalas, & Vlachostergios, 2011; Thilakarathna, McElroy, Chapagain, Papadopoulos, & Raizada, 2016).

In the intercropping of legumes with nonlegumes, N fixed by legumes becomes available to nonfixing companion species (Carranca, Torres, & Madeira, 2015; Moyer‐Henry, Burton, Israel, & Rufty, 2006). Previous intercrop study demonstrated that 32%–58% of the N assimilated by sorghum was derived from soybean (Chu, Shen, Li, Zhang, & Wang, 2004; Fujiu et al., 1990), and these N transfers appeared mostly in different communities between N_2_‐fixing and non‐N_2_ fixing plants (Hamel, Barrantes‐Cartin, Furlan, & Smith,^1991^; Chu et al., 2004; Haby, Stout, Hons, & Leonard, 2006; Isaac et al., 2012). Inoculating with arbuscular mycorrhizal fungi (AMF) and rhizobium could improve the N fixation efficiency of legumes and enhance the transfer of N to companion graminaceous crops (Fustec, Fabien, Stéphanie, & Jean‐Bernard, 2010).

Southwest China is one of the largest karst regions (0.54 million km^2^), which is fragile and easily impacted by human disturbance (Yuan, 2003). Over the past three decades, N loss from the sloping field to fissure groundwater has been accelerated because of soil aggregate breakage due to overcultivation perturbation (Du, Pan, Li, Hu, & Wang, 2011). Total N‐deficient in karst soil layer caused by N loss and low soil volume is considered to be the most important factors that influenced crop production (Qi, Wang, & Zhang, 2013). Soybean and maize are two main grain crops in the karst lands of SW China, which cultivated area is about 75% and grain products are about 82% of the crop yield. N deficiency occurs in over 80% of karst soil of SW China, which restricted regional agricultural production (Peng, Wang, Song, Zeng, & Wang, 2008). Although there is abundant research on soybean/maize, soybean/cotton, and soybean/wheat in nonkarst region (Meng et al., 2015; van Kessel, Singleton, & Hoben, 1985), the knowledge of plant–soil interaction, site classification, and crop nutrition in karst soils, particularly where N fixation by one plant component to supplant inorganic N application in the karst regions is still unknown. Therefore, it was needed to determine the rate of N fixation and transfer in soybean/maize intercropping in the Chinese karst region.

In this study, a soybean/maize intercropping in SW Chinese karst soils used ^15^N isotropic tracing was used to determine the rate of N fixation by soybean and the proportion of N fixed that was transferred to the companion species. The study included the treatment of a 30‐μm membrane separating roots of crop species yet permitting evaluation of N transfer in soil solutes and hyphae contact. The objectives of our study were to calculate the amount of N that are transferred between maize and soybean in karst soil of SW China.

## 2 MATERIAL AND METHODS

### 2.1 Experimental design

**Figure 1:**
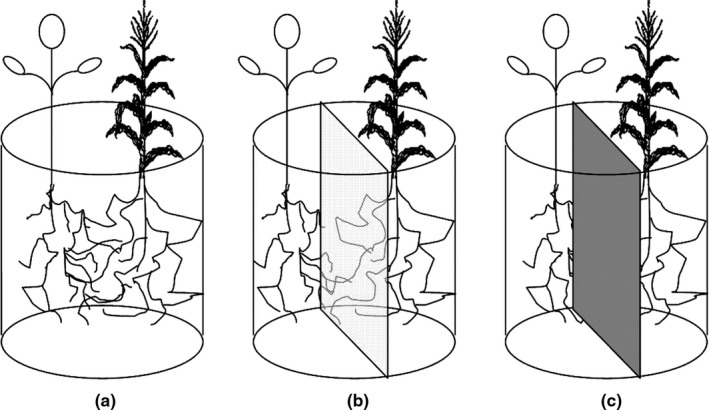
Diagrammatic drawing of three root separation pattern in this study. (a) no barrier, (b) mesh barrier, and (c) solid barrier

Pot experiments in the greenhouse were conducted from May 30 to July 28 in 2014 and 2015 at the Huangjiang karst experimental station in Huanjiang County, northwest Guangxi Zhuang autonomous region, China (24°44′–25°33′N; 107°51′–108°43′E, altitude 220 m). The soil pH was 6.83 and contained 20.19 g/kg organic matter, 1.04 g/kg total N, 0.98 g/kg total P, 49.9 mg/kg available N, and 5.65 mg/kg available P. Three pots (50 cm radius and 60 cm high) were used in this experiment (Figure 1). To simulate with the less soil depth in karst slope field, the soil was filled with top 20 cm and some rock with the below 40 cm. The experiment was a factorial design with five levels of nitrogen (N) supply: 0 (N1), 150 (N2), 300 (N3), 450 (N4), and 600 (N5) kg/ha with eight replicates. Urea fertilizers (N, 46.65%) were applied evenly over the pots before sowing. To control the soil water, the each pot was irrigated equivalently. The other field managements were conducted by the local practice of crop cultivation.

Three root separation patterns were devised between soybean and maize (Figure 1): (1) No barrier (S1), complete contact between the roots of soybean and maize was permitted; (2) mesh barrier (S2), a 30‐μm nylon mesh was used to keep roots of the intercrops separate, but permit the passage of soil solutions and contact between root hyphae; (3) solid barrier (S3), a hard plastic sheet (0.5 mm) was used to separate roots of the intercrops and prevent all transfer of solutions and hyphae. Micropots were cut in the middle, separated into three compartments, and then reconstructed for solid barrier, mesh barrier, and no barrier patterns.

Seeds of soybean (*Glycine max* L. “Guichun No. 1”) and maize (*Zea mays* L. “Xiangyu No. 7”) were surface sterilized by immersion in 10% H_2_O_2_ for 30 min before seeding. For intercropping, two seeds of maize were sown into one compartment of the pot on 30 May 2014 and 2015, and four seeds of soybean were sown into the other compartment on June 5. Once germinated, maize seedlings were thinned to one plant and soybean seedlings thinned to two plants per compartment for further growth.

Following pod initiation in soybean, we introduced labeled N using (^15^NH_4_)_2_SO_4_ (Guangzhou Puen Scientific Instrument Co. Ltd, China) enriched with 99% ^15^N. To prevent contamination from isotopic N, before labeling, we inserted a PVC board between soybean and maize shoots and set a plastic film with two layers of filter paper on top on the surface of soil. Then, a micro injector (25 μl) was used to inject 10 μl of 88 mM (^15^NH_4_)_2_SO_4_ solution into the petioles of soybeans for 9 days, with each labeling replicated four times. The plants without labeling were used as a control to examine the natural ^15^N abundance.

### 2.2 Sampling and measurements

On 28 July 2014 and 2015, we harvested the crops and separated them based on different N and root separation treatments. The biological yield of maize and bean was harvested before full maturation of seeds. Coarse roots (>2 mm) were excavated and washed with running tap water. Fine roots (≤2 mm) around crops at 0–30 cm depths were sampled using a soil corer and washed. In addition, we did not check the root nodulation in the root harvest. After killing enzymatic activity by heating to 105°C for 0.5 hr, seeds, shoots, and remaining fresh roots were dried at 70°C to a constant weight. Total N content of plants was measured using the Kjeldahl procedure, and the ^15^N abundance of shoots was determined using a DELTA PLUSXP isotope ratio mass spectrometer (FINNIGAN).

### 2.3 Data analysis

Nitrogen transferred from the ^15^N labeled soybean to the associated maize with three root separation patterns under five nitrogen (N) levels were compared using one‐way analysis of variance (ANOVA), followed by the least significant difference (LSD) method of testing differences. Significance levels were set at α = 0.05 for all statistical analyses. Moreover, this experiment is three factors including two crops, three root separations, and five N supply levels, and these data were also analyzed using multifactors analysis of variance (ANOVA). Statistical analysis was performed using SPSS19.0 software (SPSS Inc., Chicago, IL, USA).

In addition, we calculated N transfer as follows:

(1) $$N_{t}\;=\;N_{t}\%\;\times \;N_{s}\;=\;\left(\frac{N_{m}\;\times \;N_{m}\%}{N_{m}\;\times \;N_{m}\%\;+\;N_{s}\;\times \;N_{s}\%}\;\times \;100\right)\;\times \;N_{s}$$

where the different components of equation as, N_*t*_ amount of N that soybean transferred to maize (mg per plot); N_*t*_% the percentage of N uptake of associated maize after soybean transfer; N_*m*_ the N uptake of maize (mg per plot); N_*s*_ the N uptake of soybean (mg per plot). N_*m*_% and N_*s*_% indicate the atomic percentage of ^15^N excess in maize and soybean, respectively, which were computed by the difference of atomic percentage of ^15^N between the labeled plant and control plant.

(2) $$N_{o}\%\;=\;\frac{N_{t}}{N_{m}}\;\times \;100$$

where N_*o*_% indicates the percentage of transferred N from soybean that occupies the maize N uptake.

## 3 RESULTS

**Figure 2:**
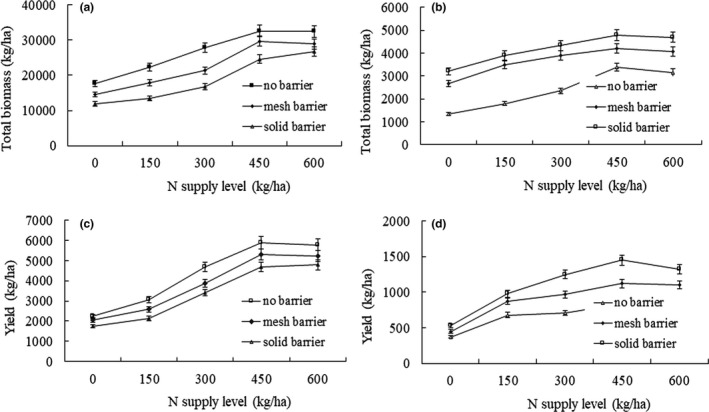
Maize biomass (a), soybean biomass (b), maize yield (c), and soybean yield (d) in soybean/maize intercropping system under different N supply levels

At all levels of N supply, biomass and yield of maize were higher when the roots were not separated (Figure 2). At N supply of 300 kg/ha, maize biomass exceeded that of the N1 by 58.5% with no root barrier, 47.1% with the mesh barrier, and 41.1% with the solid barrier. Further, N supply of 300 kg/ha significantly increased maize yield over N1 by 110% with no root barrier, 89.8% with mesh barrier, and 94.9% with solid barrier (Figure 2). Biomass and yield of soybean plants were the highest when the roots were separated, regardless of N level of applied. N supply of 300 kg/ha increased soybean biomass over N1 by 75.4% with no root barrier, 46.1% with mesh barrier, and 35.0% with solid barrier. N supply of 300 kg/ha significantly increased soybean yield over N1 by 91.9% with no root barrier, by 116% with mesh barrier and by 136% with solid barrier (Figure 2).

**Figure 3:**
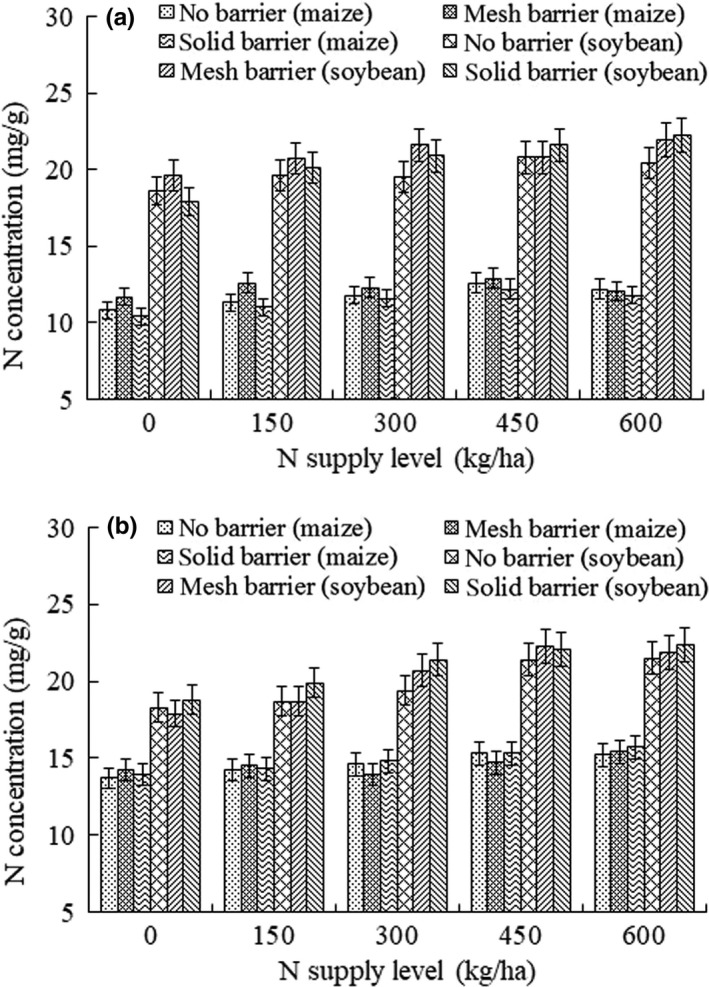
Nitrogen concentration (mg/g) of shoot (a) and root (b) in soybean/maize intercropping systems under five different N supply levels (0, 150, 300, 450, 600 kg/ha)

The shoot N concentration in maize and soybean under different N supply levels ranged from 10.4 to 12.9 mg/g and from 17.9 to 22.2 mg/g, respectively (Figure 3). Root N concentration in maize and soybean under five different N treatments ranged from 13.7 to 15.7 mg/g and from 17.9 to 22.4 mg/g, respectively. In addition, both root separation patterns and N supply showed no significant effects on N concentration in soybean/maize intercropping systems.

**Figure 4:**
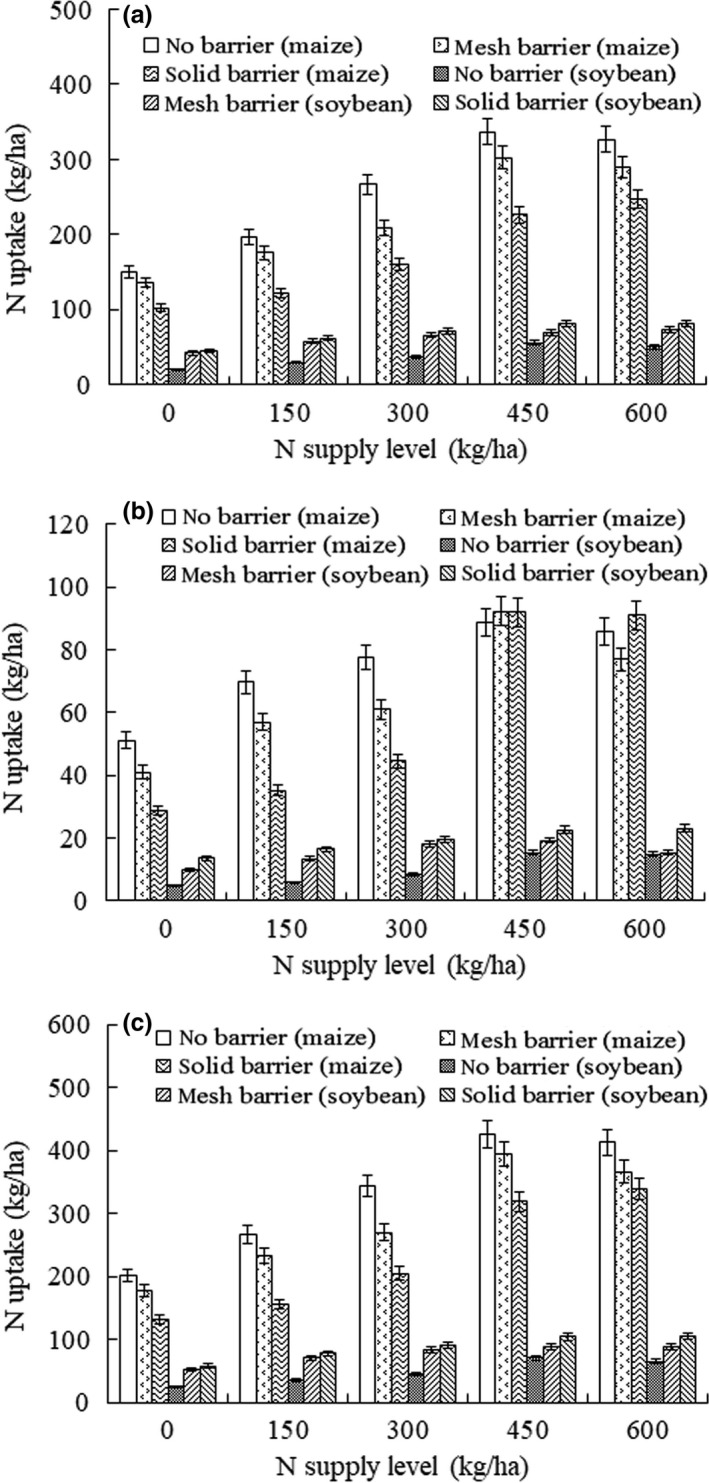
Shoot (a), root (b), and total plant (c) N uptake (kg/ha) in soybean/maize intercropping system under five different N supply levels (0, 150, 300, 450, 600 kg/ha)

At the same N supply, significant differences were observed in the N uptake of maize shoots and roots among the different root separation patterns (Figure 4). Similar significant differences were also observed in the N uptake of soybean shoots and roots among the different root separation patterns. Moreover, the N supply had a significant effect on the N uptake in soybean/maize intercropping systems. In addition, total N uptake of maize with the no barrier pattern was 14.2% higher than that for the mesh barrier pattern in N1, which showed that the N uptake of maize was significantly enhanced by intercropping with soybean.

The amount of N that soybean transferred to maize (N_*t*_) increases according to N supply (Table 1). The N_*t*_ in no barrier and mesh barrier under N1 were 8.9 and 6.9 mg per plot, respectively, while the N_*t*_ in no barrier and mesh barrier under N5 were 21.7 and 19.1 mg per plot, respectively. In addition, N transferred from soybean to maize account for 2.4%–3.3% of the N uptake of maize. However, the method of root separation did not affect the percentage of transferred N from soybean that occupies the maize N uptake (N_*o*_%). N_*t*_ with no root separation was 13.2%–30.2% more than that with mesh barrier. In addition, our results showed that biomass, yield, N concentration in plant components, N uptake, and N transferred from soybean to maize were influenced by root separation under different N supply levels (Tables 2, 3, 4). Therefore, these results suggested that N uptake and transfer in soybean/maize intercropping system were modulated by different root separations and N supply levels.

**Table 1:** Nitrogen transferred from the ^15^N labeled soybean to the associated maize with three root separation patterns under five nitrogen (N) levels

N levels (kg/ha)	N_*t*_ %		N_*t*_ (mg/plot)		N_*o*_%	
	No barrier	Mesh barrier	No barrier	Mesh barrier	No barrier	Mesh barrier
0	4.43aA	3.87aA	8.92aA	6.85aA	2.59aA	2.40aA
150	4.25aA	3.95aA	11.31aA	9.17bA	2.48aA	2.45aA
300	4.66aA	5.06bB	16.02bA	13.63cB	2.89aA	2.77aA
450	5.30bB	5.07bB	22.58cB	19.94dC	3.34bB	3.19bB
600	5.26bB	5.21bB	21.74cB	19.09dC	3.27bB	3.22bB

N_*t*_, amount of N that soybean transferred to maize (mg per plot); N_*t*_%, the percentage of N uptake of associated maize after soybean transfer; N_*o*_%, the percentage of transferred N from soybean that occupies the maize N uptake. Uppercase and lowercase letters represent *p *< 0.01 and *p *< 0.05, respectively.

**Table 2:** Results of two factorial ANOVAs for the effect three root separations and five N supply levels on the variance of maize biomass, yield, nitrogen concentration, shoot N uptake, root N uptake, total plant N uptake

Source	Biomass			Yield			Nitrogen concentration		
	*df*	MS	*F*	*df*	MS	*F*	*df*	MS	*F*
Root separation (RS)	2	56.2	132.8**	2	186.2	105.3**	2	132.7	122.9**
N supply levels (NS)	4	70.4	112.5**	4	85.7	107.9**	4	116.0	140.5**
RS × NS	8	19.2	23.4*	8	35.3	24.2*	8	13.5	5.1
Error	30	6.9		30	12.6		30	4.3	

Source	Shoot N uptake			Root N uptake			Total plant N uptake		
	*df*	MS	*F*	*df*	MS	*F*	*df*	MS	*F*
Root separation (RS)	2	101.2	139.3**	2	95.7	107.8**	2	91.7	114.2**
N supply levels (NS)	4	143.7	156.0**	4	65.9	52.4*	4	63.5	82.5**
RS × NS	8	12.5	9.1	8	17.2	13.7	8	8.7	11.3
Error	30	2.7		30	1.2		30	2.5	

For each variable, the mean square (MS) and *F*‐value (*F*) are shown.

*p < 0.05, **p < 0.01.

**Table 3:** Results of two factorial ANOVAs for the effect three root separations and five N supply levels on the variance of soybean biomass, yield, nitrogen concentration, shoot N uptake, root N uptake, total plant N uptake

Source	Biomass			Yield			Nitrogen concentration		
	*df*	MS	*F*	*df*	MS	*F*	*df*	MS	*F*
Root separation (RS)	2	83.4	115.0**	2	120.7	132.4**	2	155.2	139.3**
N supply levels (NS)	4	92.5	109.3**	4	75.8	119.4**	4	96.1	145.8**
RS × NS	8	24.8	26.1*	8	54.7	32.5*	8	34.2	22.6
Error	30	12.1		30	9.4		30	11.7	

Source	Shoot N uptake			Root N uptake			Total plant N uptake		
	*df*	MS	*F*	*df*	MS	*F*	*df*	MS	*F*
Root separation (RS)	2	152.5	148.3**	2	155.4	132.8**	2	107.9	132.5**
N supply levels (NS)	4	119.8	124.5**	4	95.4	84.3*	4	78.1	35.5*
RS × NS	8	34.5	11.9	8	29.5	17.1	8	12.8	15.3
Error	30	7.7		30	10.3		30	5.6	

For each variable, the mean square (MS) and *F*‐value (*F*) are shown.

*p < 0.05, **p < 0.01.

**Table 4:** Results of two factorial ANOVAs for the effect three root separations and five N supply levels on the variance of amount of N that soybean transferred to maize (N_*t*_, mg per plot), the percentage of N uptake of associated maize after soybean transfer (N_*t*_%) and the percentage of transferred N from soybean that occupies the maize N uptake (N_*o*_%)

Source	N_*t*_			N_*t*_%			N_*o*_%		
	*df*	MS	*F*	*df*	MS	*F*	*df*	MS	*F*
Root separation (RS)	2	103.7	128.6**	2	268.5	150.7**	2	131.2	138.5**
N supply levels (NS)	4	91.5	151.2**	4	97.6	109.2**	4	96.1	58.3*
RS × NS	8	29.6	24.1*	8	13.2	12.7	8	15.0	32.6*
Error	30	1.2		30	5.9		30	4.7	

For each variable, the mean square (MS) and *F*‐value (*F*) are shown.

*p < 0.05, **p < 0.01.

## 4 DISCUSSION

Our data indicated that maize growth in intercropping is the highest when the roots of maize have access to roots or indirect access via solutes from the hyphae of companion soybeans regardless of N supply. This confirms the yield advantage in soybean/maize intercropping systems in the karst regions of southwest China. In Huang‐Huai‐Hai Plain, China, the total grain yield (wheat + maize) of a winter wheat/spring maize intercropping system increased by 39% and 98%, respectively (Zhang et al., 2007). In northeast China, soybean and maize biomass increased by 21.7% and 16.3%, respectively, after inoculating with rhizobium in intercropping systems in reclaimed black soil (Fang et al., 2009). These illustrate that intercropping can enhance grain yield in nonkarst regions, which is consistent with our results in karst regions.

Compared to monoculture systems, in intercropping systems, crop yield improvement may be due to the greater dry matter production in maize resulting from better use of biophysical resources in strip intercropping treatments (De Medeiros, Arruda, & Sakai, 2005). Some research found that increased light capture, more efficient light use, or a combination of these two were the main reason for the greater productivity of intercropped systems relative to sole crops (Keating & Carberry, 1993; Wang et al., 2015). However, we did not measure the light use in this experiment. Thus, some future researches on the light use of intercropped systems in karst region are still used to determine whether higher radiation‐interception efficiency contribute to the yield advantage of intercropped soybean and maize in this region. Moreover, atmospheric nitrogen can be fixed using legumes, which may then be used by a host plant (Antunes et al., 2006; Bardgett, Denton, & Cook, 1999). Plants can take up N when it is released from the nodules into the soil. Our result also showed that N concentration and uptake changed with the N supply levels and root separation patterns, which imply that the different responses between soil fertility and within intercropping systems are strongly associated with feedback regulation of N absorption capacity of roots by shoot growth under nonlimiting or limiting N supply (Li et al., 2001).

Previous studies indicated two possible pathways for N transfer among intercropping species. The direct pathway transfers N fixed by legumes to associated non‐N_2_ fixing plants via a mycorrhizal fungal hyphae network (Cardoso & Kuyper, 2006; Sierra & Nygren, 2006). The indirect pathway is the release N into the rhizosphere by residual and root exudates of legumes when they decompose, and the mineralized inorganic N can then be absorbed by the intercropped graminaceous or mycorrhizal hyphae (He, Critchley, & Bledsoe, 2003; Johansen & Jensen, 1996). Our results also demonstrated direct N transfer under no root separation treatment. Additionally, although the mesh screen prevented direct contact of the roots of soybean and maize, some research demonstrated that the hyphae may penetrate the screen providing opportunities for direct N transfer (Chu et al., 2004; Li et al., 2016). In karst region, a variety of micro‐geomorphological habitats may promote the development of mycorrhizal fungal, which is closely linked to plant community and soil nutrients (Li et al.^2001^). Hence, irrespective of the manner in which N is transferred, the important role of hyphae in N transfer from soybean to associated maize may need to be mentioned in further research. Moreover, some research reported that nitrogen transfer from legumes to nonlegumes is modulated by a wide range of abiotic factors (light, water stress, soil available N, and temperature) and biotic factors (root contact, root herbivores, growth stage, plant density, defoliation, and production year; Frankow‐Lindberg & Dahlin, 2013; Meng et al., 2015). Our data demonstrated that N transfer was influenced by root separation and soil N levels, which is consistent with previous research. Finally, we also noticed that the water use, genotypic traits, and management practices may influence the crop performance in karst region. In the future research, the integrated model should be developed and used as a research tool to optimize the system management for intercropped system.

## 5 CONCLUSION

Two‐year plot experiment in the greenhouse demonstrated that mesh barrier and no restrictions barrier root separation increased N uptake of maize by 1.28%–3.45% and 3.2%–3.45%, respectively. N uptake by soybean with no restrictions root separation was 1.23 and 1.56 times higher than that by mesh and solid barriers, respectively. N transfer from soybean to maize in no restrictions barrier was 2.34‐3.02 mg higher than that of mesh barrier. Soybean/maize intercropping could improve N uptake and transfer efficiently in the karst region of southwest China.

## CONFLICT OF INTEREST

None declared.

## AUTHOR CONTRIBUTIONS

Hao Zhang and Fu ping Zeng designed the study. You zhi Li and Zhi gang Zou performed the data collection and analysis. Hao Zhang, Zhen qian Zhang, and Zhi gang Zou interpreted the results and wrote the manuscript. All authors reviewed the manuscript.
